# Genomic Origin and Diversification of the Glucosinolate MAM Locus

**DOI:** 10.3389/fpls.2020.00711

**Published:** 2020-06-04

**Authors:** R. Shawn Abrahams, J. Chris Pires, M. Eric Schranz

**Affiliations:** ^1^Division of Biological Sciences, University of Missouri, Columbia, MO, United States; ^2^Biosystematics Group, Wageningen University, Wageningen, Netherlands

**Keywords:** glucosinolates, brassicaceae, gene family, polyploidy, gene duplication, gene fusion

## Abstract

Glucosinolates are a diverse group of plant metabolites that characterize the order Brassicales. The *MAM* locus is one of the most significant QTLs for glucosinolate diversity. However, most of what we understand about evolution at the locus is focused on only a few species and not within a phylogenetic context. In this study, we utilize a micro-synteny network and phylogenetic inference to investigate the origin and diversification of the *MAM*/IPMS gene family. We uncover unique *MAM*-like genes found at the orthologous locus in the Cleomaceae that shed light on the transition from *IPMS* to *MAM*. In the Brassicaceae, we identify six distinct *MAM* clades across Lineages I, II, and III. We characterize the evolutionary impact and consequences of local duplications, transpositions, whole genome duplications, and gene fusion events, generating several new hypothesizes on the function and diversity of the *MAM* locus.

## Introduction

Glucosinolates (GSL) are a diverse class of amino-acid derived sulfur containing metabolites characteristic of plants of the order Brassicales (Rodman et al., 1998; Borpatragohain et al., 2016; Kliebenstein and Cacho, 2016; Olsen et al., 2016; Chhajed et al., 2019; Blazevic et al., 2020). When the plant experiences physical damage, such as chewing by herbivores, compartments of the cell rupture and release myrosinase enzymes that hydrolyze the GSLs to create an isothiocyanate anion, damaging the attacker (Rodman et al., 1998). Besides their roles in direct defense, GSLs have also been shown to play important roles such as nutrient transport and physiological signaling (del Carmen et al., 2013). They are considered a key innovation of the Brassicales, as adaptations in the biosynthesis pathway have been shown to correlate with increased rates of speciation (Edger et al., 2015). The GSL pathway is a model for investigating processes underlying natural variation within and among species; including the roles of genome and gene duplication (Kliebenstein, 2008; Bekaert et al., 2012; Hofberger et al., 2013; Edger et al., 2015; van den Bergh et al., 2016; Wisecaver et al., 2017). Aliphatic GSLs, the largest sub-group of compounds, are especially implicated in this rate of speciation as they are only found in the most species-rich groups such as the family Brassicaceae.

The often multi-gene *methylthioalkylmalate* (*MAM*) locus, also called the Elong locus, accounts for much of the natural variation observed in aliphatic GSLs (Kliebenstein et al., 2001a, b; Textor et al., 2004, 2007; Kroymann and Mitchell-Olds, 2005; Benderoth et al., 2006, 2009; Keurentjes et al., 2006; de Kraker et al., 2007; Wentzell et al., 2007; de Kraker and Gershenzon, 2011; Zhang et al., 2015; Kliebenstein and Cacho, 2016; Kumar et al., 2019; Petersen et al., 2019). *MAM* enzymes catalyze the condensation reaction that extends the carbon chain in amino acid derived GSL precursors (Benderoth et al., 2006). The extended amino acid expands the types (Kliebenstein and Cacho, 2016). Most of what we understand about the evolution of *MAM* has been learned from studying just a handful of species, without a broad phylogenetic context (Kliebenstein and Cacho, 2016). MAM diversification in the Brassicaceae is thought to have occurred independently in separate lineages. Specifically, *MAM* diversity has been largely examined in Lineage I of the family (*Arabidopsis* and relatives) and to a lesser extent in Lineage II (*Brassica* and relatives). This work has been supported by large gene datasets, though with differing gene tree topologies (Zhang et al., 2015; Supplementary Figure S1).

In *Arabidopsis thaliana*, phenotypic variation of the *MAM* locus is characterized by the accumulation of different majority carbon chain-length GSL profiles (Kliebenstein and Cacho, 2016). The most common profiles have majority three carbon (3C) or four carbon (4C) molecules, but can extend up to 8C majority profiles, with variability at the population level (Benderoth et al., 2009; Kliebenstein and Cacho, 2016). Copy number variation and allelic diversity/presence-absence drive these differences, as one *MAM* gene may mask the phenotype of another at the same locus (Benderoth et al., 2006, 2009). This plays out in the interactions between *MAM1* and *MAM2* in *A. thaliana* populations, where variation is well understood. The 4C majority phenotype is seen in populations where *MAM1* and *MAM2* are both present and intact or when *MAM2* is absent. In populations lacking a *MAM1* gene, the GSL profile exhibits a 3C majority phenotype. In some cases, *MAM1* and *MAM2* genes have been fused (e.g., gene chimerism) wherein they are reformed into a *MAM1-like* functional gene with partial *MAM2* sequences, or vice versa (Benderoth et al., 2006). Crop Brassicas most commonly accumulate 3C, 4C, or a mix of 3C and 4C majority profiles, the latter displaying a seemingly unmasked phenotype, unlike what we see in *A. thaliana* (Benderoth et al., 2009; Kliebenstein and Cacho, 2016).

Naming conventions for *MAM* orthologs are either directly based on *A. thaliana* (*MAM1*, *MAM2*, and *MAM3*) or based on *A. lyrata MAM* (*MAMa, MAMb*, and *MAMc*) (Benderoth et al., 2009). The *Arabidopsis* centered model of *MAM* diversity is vulnerable to miss-characterization as *Arabidopsis* genes may be highly derived, and thus not generalizable. We also see that the number of genes at the *MAM* locus can vary between populations as well as species, potentially misleading ancestral state estimations with poor sampling. To accurately understand *MAM* diversification, it is necessary for gene selection across a broader species phylogeny with comparisons to their primary metabolic ancestor, isopropylmalate synthase (*IPMS*).

Though diverged, *IPMS* and *MAM* share a high sequence similarity and similar enzymatic function (Moghe and Last, 2015). I*PMS* contains two conserved protein domains: a pyruvate carboxylase (HMGL-like), that is involved in the carbon condensation reaction, and a leucine allosteric domain (LeuA), that commits the protein to the leucine biosynthesis pathway forming a homodimer (Koon et al., 2004). *MAM* genes only retain the HMGL-like domain, the loss of LeuA being considered a key step in the transition of *MAM* from an *IPMS-like* gene (de Kraker et al., 2007). To our knowledge, no previous work has investigated when the loss of this domain occurred in the evolution of the locus.

In this study, we examine the evolutionary history and diversity of the *MAM/IPMS* gene family, uncovering critical steps in the origin of *MAM* and identifying patterns of domain-specific diversity across the Brassicaceae and its sister-family the Cleomaceae. We utilize a genomic networking methodology to analyze the wealth of newly available genome sequences (Zhao et al., 2017; Zhao and Schranz, 2019). The method analyses the conserved physical location of gene family members across queried genomes, known as synteny, to characterize the impact of different gene duplication types in the expansion of the *MAM/IPMS* gene family (Zhao et al., 2017; Zhao and Schranz, 2019). Ultimately we show that a mix of gene duplication types and domain changes played important roles in the evolution and innovation of the *MAM* locus.

## Materials and Methods

### Genomic Network Construction

The genomic network analysis included 40 complete plant genomes representing 38 different species. This included 34 Brassicaceae species from Lineages I, II, III, and *Aethionema arabicum* as sister to the rest of the family, three genomes from the sister-family Cleomaceae, and three outgroup species (*Theobroma cacao, Citrus sinensis, and Vitis vinifera*) (Supplementary Table S1). For each genome, we utilized protein sequences in FASTA format and a BED/GFF file. One of two *Capsella rubella* genomes was excluded from downstream analysis due to insufficient quality. The *Thellungiella halophila* and *Thellungiella salsuginea* are two different sequencing efforts of the same species, now under the name *Eutrema salsugineum*. The genome sequenced as *Alyssum linifolium* has since been identified as *Descurainia pinnata*. Network analyses were performed as described in Zhao et al. (2017). Reciprocal all-against-all whole genome protein sequence comparison were made using RAPSeach2 (Zhao et al., 2012). MCScanX (Tang et al., 2008; Wang et al., 2012) was used to calculate generic collinearity between genomes and all comparisons were saved to generate the full genomic network.

### Gene Family Network

We identified candidate *IPMS/MAM* genes using HMMER (Finn et al., 2011), cross-referencing the Pfam, PDBe, and GO databases with domain signature HMGL-like PF00682, and filtered by an inclusion threshold e-value of 0.007. Selected genes were later filtered by relative branch lengths as compared to known *IPMS* and *MAM* genes and then queried against the overall syntenic network with a 25 gene window to extract the gene family network. We visualized the resulting network in Cytoscape version 3.3.0 (Shannon et al., 2003). We then pruned the network of gene nodes that did not contain an HMGL-like domain but were dragged in by potential domain fusions. Clique percolation, as implemented in CFindier (Derényi et al., 2005; Palla et al., 2005; Fortunato, 2010), was used to locate all K-clique comments to identify communities or clusters of gene nodes.

### Phylogenetic Inference

Full amino acid sequences for all gene family members were aligned using MAFFT (Kuraku et al., 2013; Katoh et al., 2017) and cleaned using Phyutility at a 50% occupancy threshold (Smith and Dunn, 2008). We used RAxML (Stamatakis, 2014) for phylogenetic inference with the GTRCAT model (Boostrap = 1000). The same procedure was repeated for the HMGL-like domain region of each gene FASTA file as estimated by HMMER. Supplemental sequence comparisons were made using MView (Madeira et al., 2019) and analyzed using R.

## Results

### Synteny and Domain Analysis

**FIGURE 1:**
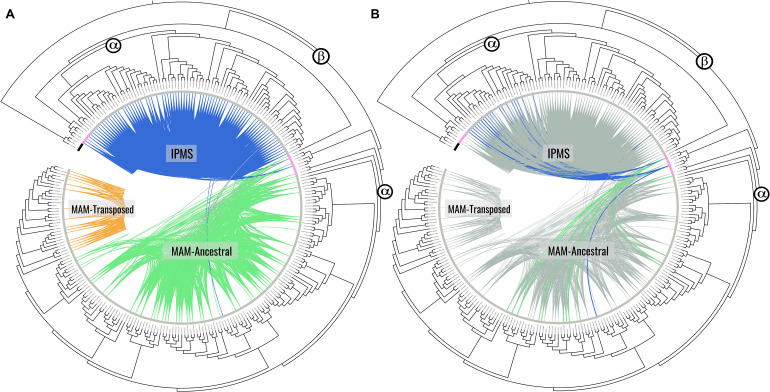
Synteny clusters and gene tree phylogeny of identified *IPMS* and *MAM* genes consisting of 262 total. For **(A,B)** the bar along the tips represent species lineage where black bars indicate genes from out group genomes, the pink bar indicates genes from Cleomaceae genomes, and the gray bar indicates genes from Brassicaceae genomes. **(A)** Syntenic cluster analysis identified three distinct gene clusters, each representing a different conserved genomic location. The IPMS cluster in blue, the MAM-Ancestral cluster in green, and the novel lineage specific MAM-Transposed cluster in orange. Gray lines here indicate connections between the IPMS and MAM clusters. **(B)** Emphasizes those connections between MAM-like genes in the Cleomaceae that exhibit both *IPMS* & *MAM* cluster membership (Clevi.0004s0713 and tha_Th2v24105) despite being physically located at the MAM-Ancestral locus in their respective genomes. [For Bootstrap scores: Supplementary Figure S6; Online interactive trees: **(A)** http://bit.ly/2tHVgYK; **(B)** http://bit.ly/2Svu8Vf].

Micro-synteny network analysis identified three major syntenic clusters (Figure 1), two of which encompass many genes of the known *MAM* gene clade (orange and green clusters) and one encompassing the known *IPMS* gene clade (blue cluster). Of the syntenic clusters found in the *MAM* clade, the green cluster identifies the ancestral *MAM* position, what we will call the *MAM*-Ancestral locus, and is equivalent to the Elong locus. The orange cluster represents a transposed and retained *MAM* locus-specific to Lineage II of the Brassicaceae, which we will call the *MAM*-Transposed locus. The analysis also recovered the 4th cluster of an unnamed lineage of genes that have retained only a single HMGL-like domain and are found in both our outgroup and in-group genomes. The *A. thaliana* representative gene of this clade (AT2G26800) has been shown to play a role in seed amino acid concentration (Peng et al., 2015). Relative branch lengths showed this gene clade as highly diverged from both MAM and IPMS sequences. Because of this, all genes of this clade were filtered from downstream analyses.

95.7% of IPMS genes identified by sequence were also found in the IPMS syntenic cluster. 39.6% of MAM genes, not associated with the conserved Lineage II transposition, were found in the MAM-Ancestral syntenic cluster. 51.6% of genes found in the Lineage II transposed sub-clade were found in the syntenic cluster. Differences in percent synteny are tied to increased rates of tandem duplications, as the local duplicate syntenic signal was often masked, and transposed duplication events, which remove syntenic context. It is expected that many new transposed duplicates are in the process of pseudogenization and are not active MAM genes.

**FIGURE 2:**
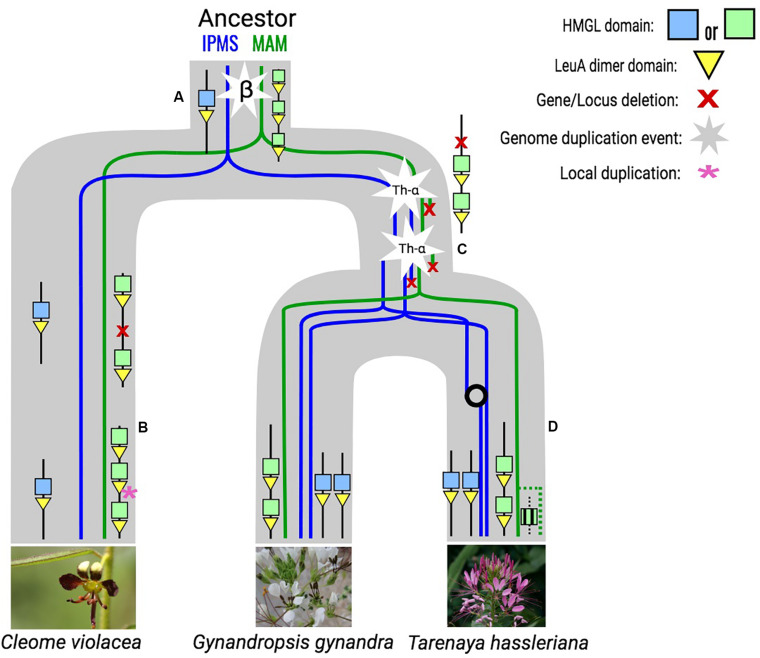
Inferred evolutionary trajectory of *MAM* and *IPMS* loci in the cleomaceae based on genomic synteny and phylogenetic information. **(A)** The MAM-Ancestral locus originated from the β whole genome duplication event and is characterized by *MAM*–like genes that experience local duplication and have retained their LeuA domain. **(B)** In the genome of *Cleome violacea* there is a gene deletion followed by a novel tandem duplication at the MAM locus. **(C)** Following the Th-α whole genome triplication the *IPMS* locus is duplicated and the *MAM* locus experiences compensatory gene loss and is reflected in the *Gyanandropsis gynandra* genome. **(D)** In the *Tarenaya hassleriana* genome, the IPMS locus experiences a gene conversion event that maintains sequence similarity between the two copies. There is also a novel transposition of the MAM-like gene from the MAM-Ancestral locus that does not maintain the LeuA domain. As the placement of the Th-α whole genome duplication event is not confirmed to be fully shared by both lineages, an alternative reconstruction is also possible.

All genes at the Cleomaceae *MAM*-Ancestral locus have retained their LeuA domain from their time as *IPMS* duplicates, with some showing syntenic connections to both the *MAM*-Ancestral and *IPMS* syntenic cluster (Figure 1). For example, Th2v2405 from *Tarenaya hassleriana* has more syntenic connections with *IPMS* cluster members than with genes of the *MAM*-Ancestral locus, despite belonging to the direct orthologous chromosomal region of the MAM-Ancestral locus in the Brassicaceae (Figures 1, 2). Genes of the Cleomaceae *MAM*-Ancestral locus and the IPMS locus also appear to have a shared pattern of gene dosage. A duplication of the IPMS locus following WGD, brings the total *IPMS* gene number to two, followed by a compensatory reduction in MAM gene number at the *MAM*-Ancestral locus (Figure 2C). An exception to this is found in the *Tarenaya hassleriana* genome, where a novel transposed *MAM*-like gene has lost the LeuA domain. This allows for three *MAM*-like genes to co-occur with two *IPMS* genes (Supplementary Figure S3).

### Gene Family Relationships

**FIGURE 3:**
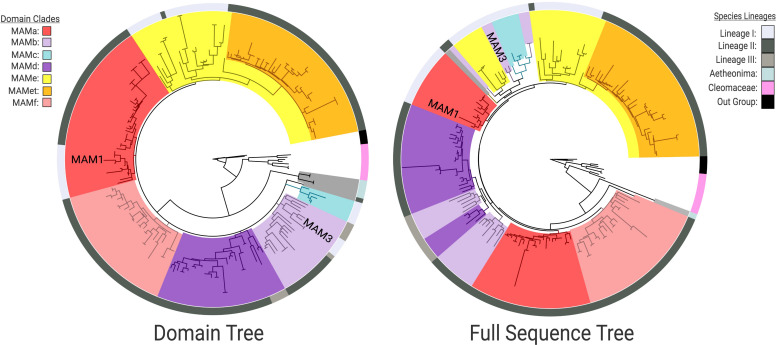
Clade comparison between Brassicaceae MAM domain and full sequence gene trees highlighting incongruence. HMGL-like sequences were used for the domain tree and resolved six clades of MAM (MAMa-f) but could not infer branching order. In the full sequence tree there is a breakdown of MAMa and MAMb that is correlated with species lineage. In both trees, the placement of MAM1 and MAM3 from the *Arabidopsis thaliana* Col genome are indicated. [For Bootstrap scores: Supplementary Figure S7 – Domain Tree; Supplementary Figure S8 – Full Sequence; Online interactive trees: Domain – http://bit.ly/2Hb5jIS; Full Sequence – http://bit.ly/37btHEZ].

The HMGL-like domain and full protein sequence gene trees identified distinct IPMS and MAM clades (Figure 1). In both cases, Cleomaceae genes are sister to a larger Brassicaceae clade, and *Aethionema arabicum* is sister to the rest of the Brassicaceae, which agrees with the species tree topology. Within the core Brassicaceae, the domain and full sequence trees display topological incongruence to each other (Figure 3) and neither perfectly match the species tree.

The domain tree divides MAM into six supported clades (Figure 3). Though the branching order could not be determined, the supported clades were assigned *MAMa-f*. These domain clade designations are based on the *Arabidopsis lyrata* MAM gene-tree clades. Given the branch length, a measure of sequence divergence, of the genes found at the MAM-Transposed locus (Figure 3), the sub-clade of *MAMe* was designated *MAMet*. The closest non-*MAMet* domain sequence to the group was a *MAMe* sequence from the *Lunaria annua* genome.

**FIGURE 4:**
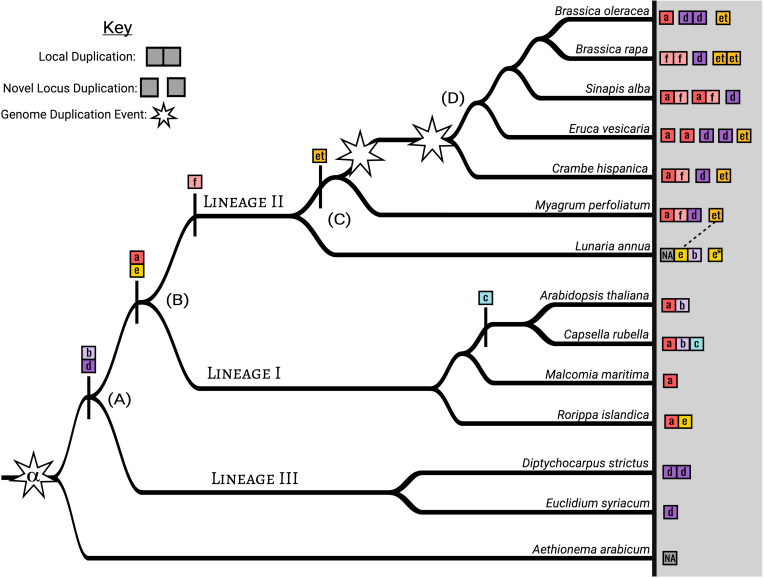
MAM clade and genomic context diversity within the Brassicaceae based on a subsample of analyzed genomes. Each square represents a MAM gene with an indicated HMGL-like domain type. Connected squares are found at the same physical location in the genome and not connected squares represent separate MAM loci (i.e., the MAM-Ancestral locus, a syntenic duplicate of the of the MAM-Ancestral locus, or MAM-Transposed). Non-syntenic gene transpositions were not included. **(A)** We estimate that the shared ancestor of Lineages I, II, and III maintained both MAMb and MAMd domain types. In the Lineage III genomes sampled MAMb genes were not located at the MAM-Ancestral locus, but at transposed loci. **(B)** At the ancestor of Lineage I and II, MAMa and MAMe appear, while the MAMc innovation occurs within a sub clade of Lineage I. **(C)** MAMf originates at the ancestor of Lineage II. The MAMet transposition that creates the MAM-Transposed locus occurs following the split from Lunaria annua, with all MAMet genes being closely related to a MAMe gene at the MAM-Ancestral locus. Lunaria annua also contains a context duplication of the MAM-Ancestral locus* that does not appear to be associated with whole genome duplication. **(D)** The unnamed whole genome duplication found in the tribe Brassiceae of Lineage II has resulted in multiple context duplications of the MAM-Ancestral locus. Full comparison is found in Supplementary Figure S3.

Summary amino acid comparison at 80% similarity threshold shows *MAMa* is the most conserved domain, *MAMe* is the most variable domain, and *MAMet* and *MAMc* are the most diverged (Supplementary Figure S5). Exon/Intron comparisons of full *MAMet* genes show the expected number of domains for a functional MAM gene but with differences in exon size. When plotted on the species tree, *MAMa-b* and *MAMe* are ancestral to Lineage I, *MAMa-b* and *MAMd-f* are ancestral to Lineage II, and *MAMb* and *MAMd* are ancestral to Lineage III (Figure 4 and Supplementary Figure S3).

The MAM full-sequence tree shows bootstrap support between clades, but also a breakdown of some domain clades as well as clade nesting (Figure 3). *MAMa* and *MAMb* separate by species lineage, while MAMc is unique to a small subset of Lineage I species and appears closely related to *MAMb* and *MAMe*. MAMd, and MAMe are primarily the same as in the domain tree, but with other domains nested within. MAMf is consistent with the domain tree and sister to Lineage II *MAMa*.

To test for potential gene fusion events, full sequences of *MAMa* and *MAMb* Lineage I genes were broken up into “before the domain,” “domain,” and “after domain” sequences (Supplementary Figure S4). Pairwise sequence comparisons were made between the Lineage I gene segments and corresponding segments of Lineage I *MAMe* genes, and Lineage II genes for *MAMa* or Lineage III genes for *MAMb*. In both cases, the domain portion best matches the corresponding domain regardless of Lineage. For Lineage I *MAMb*, the region before the domain is more similar to Lineage I MAMe than it is to Lineage II *MAMb*. For Lineage I *MAMa*, the region before the domain is more similar on average to Lineage I MAMe but was not significantly different from Lineage II *MAMa*.

## Discussion

The origin of all specialized metabolic pathways is primary metabolic genes, often with similar enzymatic chemistry (Moghe and Last, 2015). This transition is mediated by the process of gene duplication and subsequent drift and neo/subfunctionalization (Conant and Wolfe, 2008; Moghe and Last, 2015). For the MAM locus of the glucosinolate (GSL) biosynthesis pathway, the role of tandem duplication events in the evolution of the locus has been well characterized at the population level. The majority of work has only looked at Arabidopsis and its close relatives, and to a lesser extent, in the crop Brassicas (Kliebenstein and Cacho, 2016). Much of what we understand about the MAM locus function has not been understood in the context of phylogeny, except to say that based on gene tree relationships, Lineage II and Lineage II have independently diversified from some initial gene substrate (Benderoth et al., 2009; Zhang et al., 2015). In this study, we utilized a micro-synteny network of genomes and phylogenetic inference to elucidate the evolutionary history of the MAM locus.

### MAM in the Cleomaceae

The inclusion of Cleomaceae genomes in our analysis has provided novel insight into the origin of the *MAM* locus, following the whole genome duplication (WGD) event β, the hypothesized origin of *MAM* from *IPMS* (van den Bergh et al., 2016). We estimate through micro-synteny and gene tree information that the Ancestral-*MAM* locus at the formation of the Cleomaceae was characterized by multiple MAM-like gene duplicates, the result of tandem duplications or local transposition (Figure 2). These genes are different from what has been characterized in the Brassicaceae orthologous Ancestral-*MAM* locus, the Elong locus. They have retained their LeuA domain, the loss of which has been considered a critical step in the evolution of Brassicaceae MAM (de Kraker et al., 2007). Within the Cleomaceae, some genes of the Ancestral-*MAM* locus exhibit both Ancestral-*MAM* and *IPMS* syntenic cluster identity (Figure 1). The syntenic window for these intermediates is shifted in comparison to other analyzed neighboring MAM-like genes. This allows for the inclusion of neighboring non-MAM genes that are more characteristic of the IPMS genomic context. This evidence supports the hypothesis that the Ancestral-MAM locus was once a full context duplicate of the IPMS locus, and in the process of specialization over millions of years, degraded in collinearity.

How these MAM-like genes interact with GSL biosynthesis is unknown, but they have shown levels of expression in the leaf, seed, and roots in *Tarenaya hassleriana* (van den Bergh et al., 2016). The retention of the LeuA domain suggests that MAM-like proteins may have some continued interaction with IPMS or leucine biosynthesis. The ways in which genes respond to duplication events are constrained by their biochemical interactions, and therefore may shed insight into enzyme behavior (Bekaert et al., 2012; Birchler and Veitia, 2012; Conant et al., 2014; McLysaght et al., 2014). For example, given that IPMS experiences purifying selection of local gene duplicates and that *MAM-like* Cleomaceae genes found at the *MAM-*Ancestral locus do exhibit some local duplication, it is likely that these MAM-like genes have significantly sub- or neofunctionalized from their IPMS ancestor in terms of biochemical role. With that said, the dosage effects of *IPMS* are broader than only limiting local duplication, and through stoichiometric effects constrain most duplication types. Only after the β WGD event, is IPMS able to be retained and reduced in multiples of two. A pattern we see recapitulated after subsequent WGD events, with a few potential exceptions (Supplementary Figure S2). Following Th-α, the Cleomaceae whole-genome triplication (WGT) or hexaploidy, there is an expected full context duplication of the IPMS locus, but with no context duplication of the Ancestral-*MAM* locus (Figure 2C). In fact, we see a compensatory loss of a *MAM-like* gene following the increase in IPMS copy number. The presence of stoichiometric conflict between IPMS and these *MAM-*like genes would support the hypothesis that they have retained some IPMS role and constraint. Further sampling across the Cleomaceae will be necessary to see if these patterns hold.

In the *Tarenaya hassleriana* genome, there is a novel a transposition of *MAM* (Figure 2D). This transposed gene does not have a LeuA domain, bringing the overall *MAM/IPMS* gene number beyond what would be expected under an IPMS dosage constraint (Supplementary Figure S2). This transposed locus has been shown to express in several tissues and to a greater extent in the leaf when compared to *MAM*-like counterparts at the Ancestral-*MAM* locus (van den Bergh et al., 2016). Increased species sampling, as well as an understanding of population-level variation in Cleomaceae *MAM*, is necessary for any conclusions on the dosage to be explored further using these methods. Direct biochemical assays of these *MAM*-like proteins will also be critical for characterizing any role they may play in glucosinolate biosynthesis and how that may differ from what is seen in the Brassicaceae. The Cleomaceae, and potentially the Capparaceae, which also shares the β duplication event (Edger et al., 2015), could serve as a powerful window into the evolution of early Brassicaceae *MAM* and a model for how gene families transition from primary to specialized metabolism.

### MAM in the Brassicaceae

Between Lineages I, II, and III of the Brassicaceae, we have identified six distinct clades of *MAM*, *MAMa-f*, based on conserved HMGL-like domain sequences (Figure 3 and Supplementary Figure S3). Based on occurrence patterns across the family, we can say that *MAMb* and *MAMd* clades are ancestral to all three lineages, and *MAMa* and *MAMe* may be ancestral to only Linages I and Lineage II. The latter conclusion could not be confirmed by gene tree information and may be vulnerable to sampling bias. The dispute between the chloroplast and nuclear species tree topologies could also affect the evolutionary relationships between the MAM clades and hamper our ability to predict (Nikolov et al., 2019). Improved sampling across the Brassicaceae is necessary before a robust estimation of the ancestral type can be made. That said, we are confident that *MAMc, MAMet*, and *MAMf* domain types are more recent innovations occurring in Lineage I and Lineage II, with specific branch placements (Figure 4 and Supplementary Figure S3).

Given the functional role this domain plays in MAM biochemistry, we expect amino acid differences between domain types to be associated with generalizable patterns in *MAM* function. *MAMa* is the most conserved of the domains (Supplementary Figure S5B), suggesting that *MAMa* genes may contribute a necessary function to GSL biosynthesis, as compared to other MAM types. *MAMc* and *MAMet* are the most diverged, each having several unique amino acid substitutions when compared to other domain types (Supplementary Figure S5B). Across all the domains, some sites were characterized by amino acid variability within and between domain types. Based on the characterization of *MAM* proteins in *Brassica juncea* (Kumar et al., 2019), we identified that oxo-acid binding sites were most often found at flexible amino acid positions followed by COA binding sites (Supplementary Figure S5A). A better understanding of these patterns can give us insight into the forces driving the adaptation of *MAM*.

The domain and full-sequence gene trees conflict most significantly within the core Brassicaceae (Figure 3). In the full-sequence tree Lineage I *MAMa* and *MAMb* genes appear more closely related to *MAMe* genes than to other genes of their shared domain. Sequence comparison reveals split-sequence similarities in both *MAMa* and *MAMb* domain clade groups. This pattern suggests two possibilities: (1) *MAM* genes experienced convergent evolution of their amino acid sequences, or (2) a gene fusion event of separate *MAM* types occurred sometime during the divergence of Lineage I MAM. The latter scenario is both the more parsimonious conclusion, and it is supported by the previous characterization of population-level gene fusion events at the Ancestral-*MAM* locus (Benderoth et al., 2009). Given that Lineage I *MAMa* and *MAMb* genes show a close phylogenetic relationship to Lineage I *MAMe*, in conflict with the domain tree, it is the most likely donor gene. Both fusion events would have occurred at separate nodes of the Lineage I species tree, *MAMa/MAM*e fusion happening earlier than the *MAMb/MAMe* event. Improved sampling of Lineage I is necessary to identify the specific species branch points at which the events occurred. The fusion of MAM genes at the MAM-Ancestral locus, though largely studied from only a population level, may have been a critical driver of MAM diversity and innovation within Lineage I in the Brassicaceae.

Most of the genes in each domain clade exist at the *MAM*-Ancestral locus. This is true for genes of the *MAMe* group except for a nested clade of transposed genes, *MAMet*, that form the unique syntenic cluster MAM-Transposed (Figures 1, 4). There are subsequent transpositions from the MAM-Transposed locus, many of which show signs of degradation. The initial transposition occurred sometime following the split from the ancestor of *Lunaria annua* to the common ancestor of *Thellungiella (Eutrema*) and the rest of Lineage II (Supplementary Figure S3). Following the transposition event, there is a loss of all *MAMe* domain type genes. Of our dataset, *L. annua* is the only member of Lineage II to retain any copies of *MAMe*. Of those *MAMe* genes, most appear closely related to Lineage I *MAMe* genes, while one copy is most closely related to *MAMet* in both the domain and full sequence trees (Figure 3). This transposition event is the earliest conserved instance of a novel *MAM* context, which allows for an escape from cis-regulatory effects that may be experienced at the *MAM*-Ancestral locus (Chen and Ni, 2006; Conant and Wolfe, 2008). The possibilities exist that these genes are performing some yet to be characterized function or potentially may represent the GSL-PRO locus characterized in Brassica species. With this current analysis, we cannot further speculate on the role *MAMet* genes may be playing in GSL biosynthesis, except to say that experimental analysis of these genes will be necessary to understand their place in metabolic innovation.

Polyploidy offers another mechanism for *MAM diversification, by escaping* potential cis-regulatory effects of other *MAM* genes or sub- and neofunctionalization of resulting duplicates. In the Cleomaceae, the *MAM-*Ancestral locus duplicates are not retained following genome doubling, putatively due to the presence of their LeuA domain and restrictions under gene dosage. Without such dosage constraints in Brassicaceae *MAM*, most genomes sampled show retention of a duplicated *MAM*-Ancestral locus following known WGD events. For example, the WGT event in the tribe Brassiceae of Lineage II resulted in three homoeologous *MAM*-Ancestral loci in subsequently diploidized genomes (Figure 4 and Supplementary Figure S2). In *Brassica rapa*, *Brassica oleracea, and Eruca vesicaria*, the *MAM*-Ancestral loci maintain a single MAM domain type (*MAMa, MAMd, or MAMf*) at each. Whereas in other genomes, like Sinapis alba, *MAMa* and *MAMf* genes remain paired although duplicated at separate loci. We propose that phenotypic differences between Brassica and Arabidopsis, such as the ability to co-synthesize different carbon chain majority phenotypes, are facilitated by the physical separation of MAM genes within the genome. By influencing the rate of diversification for MAM genes at the different MAM-Ancestral loci and allowing for novel genomic interactions, the WGT may have been a critical step in driving the specialized metabolic innovation we see in this dynamic crop lineage.

## Conclusion

The *MAM*/IPMS gene family serves as an excellent example of how a primary metabolic gene can, over millions of years and leveraging any source of novelty, give rise to a diverse lineage of highly adaptive specialized metabolic genes. Utilizing micro-synteny gene networks and broad phylogenetic sampling, we find that multiple modes of gene duplication have significantly influenced the evolutionary trajectory of the *MAM* locus and thereby diversity of aliphatic GSL profiles. By exploring some of the evolutionary consequences of whole-genome duplication, gene transposition, local duplication, and gene fusion, we have generated several new testable hypotheses as to the nature of MAM and GSL diversity. In the future, new experimental approaches and broad phylogenetically informed sampling will be critical to continue developing a robust understanding of this important gene family.

## Data Availability Statement

The datasets generated for this study can be found in the information provided in Supplementary Material.

## Author Contributions

RSA performed research and wrote the manuscript. JCP helped design the study and edited the manuscript. MES designed the study and helped write and edit the manuscript.

## Conflict of Interest

The authors declare that the research was conducted in the absence of any commercial or financial relationships that could be construed as a potential conflict of interest.

## Supplementary Material

FIGURE S1 **(A)** Benderoth et al., 2009 describes the MAM lineage in terms of orthology to *Arabidopsis lyrata* gene tree clades. While the topology generally agrees with our tree, the emphasis on Arabidopsis and close relatives gives a limited picture of MAM diversity. This tree also supported the hypothesis that MAM has evolved separately in the Lineage I and II. **(B)** Zhang et al., 2015 generally agrees with this hypothesis though they do show shared clades not solely informed by the species tree. Some of their topology conflicts with our full sequence tree and yet agrees with the domain specific tree. This may be due to how their alignment was cleaned and their species sampling.

FIGURE S2 The overall gene counts per genome for the *MAM/IPMS* gene family. Gene numbers, especially in *IPMS*, are correlated with recent polyploidy. Three genomes conflict with the expected IPMS dosage expectation of multiples of two. The *Raphanus raphanistrum* and *Stanleya pinnata* IPMS deviations may be an artifact of lower quality genomes, but the Eruca vesicaria retention appears to be a newly sub-functionalized *IPMS* copy, exhibiting an intermediate syntenic relationships to that of some MAM-Ancestral genes in the Cleomaceae. For MAM, the number of Loci indicates whether *MAM*-Ancestral or MAM-Transposed has experienced a context duplication. The number of genes at that locus is the overall total of genes across all syntenic loci of that type.

FIGURE S3 Here we show the full domain clade distribution of MAM genes across the genomes, regardless of synteny or genomic position. This data was used ultimately to place the points of innovation for different MAM types in Figure 4.

FIGURE S4 MAM protein sequences were divided into before domain, domain, and after domain segments and each significantly different section of the MAMa or MAMb genes from lineage I were compared to corresponding MAMe sections.

FIGURE S5 Amino acid sequence comparisons at 80% sequence similarity. **(A)** Colored rectangles indicate specific biochemical functions as described by Kumar et al. (2019) in Brassica juncea. Green - metal binding sites; Yellow - catalytic sites; Red - 2- oxo acid binding sites; Blue - CoA binding sites. **(B)** Summarizes all sites with a uniquely divergent amino acid to quantify the significance of domain divergence.

FIGURE S6 Full gene family phylogeny with bootstrap scores at 1000 bootstraps with syntenic clusters mapped. Used in Figure 1. May also be accessed via: http://bit.ly/2tHVgYK.

FIGURE S7 Domain tree phylogeny with clades colored and bootstrap scores at 1000 bootstraps. Used in Figure 3. May also be accessed via: http://bit.ly/2Hb5jIS.

FIGURE S8 Full gene family phylogeny with bootstrap scores at 1000 bootstraps with clades colored. Used in Figure 3. May also be accessed via: http://bit.ly/37btHEZ.
