# Supplementary material for: Genomic Origin and Diversification of the Glucosinolate MAM Locus
Source: Front Plant Sci. 2020 Jun 4;11:711. doi: 10.3389/fpls.2020.00711 (PMC7289053; doi:10.3389/fpls.2020.00711)
Supplement: FIGURE S1 — (A) Benderoth et al., 2009 describes the MAM lineage in terms of orthology to Arabidopsis lyrata gene tree clades. While the topology generally agrees with our tree, the emphasis on Arabidopsis and close relatives gives a limited picture of MAM diversity. This tree also supported the hypothesis that MAM has evolved separately in the Lineage I and II. (B) Zhang et al., 2015 generally agrees with this hypothesis though they do show shared clades not solely informed by the species tree. Some of their topology conflicts with our full sequence tree and yet agrees with the domain specific tree. This may be due to how their alignment was cleaned and their species sampling. [file Presentation_1.pdf]

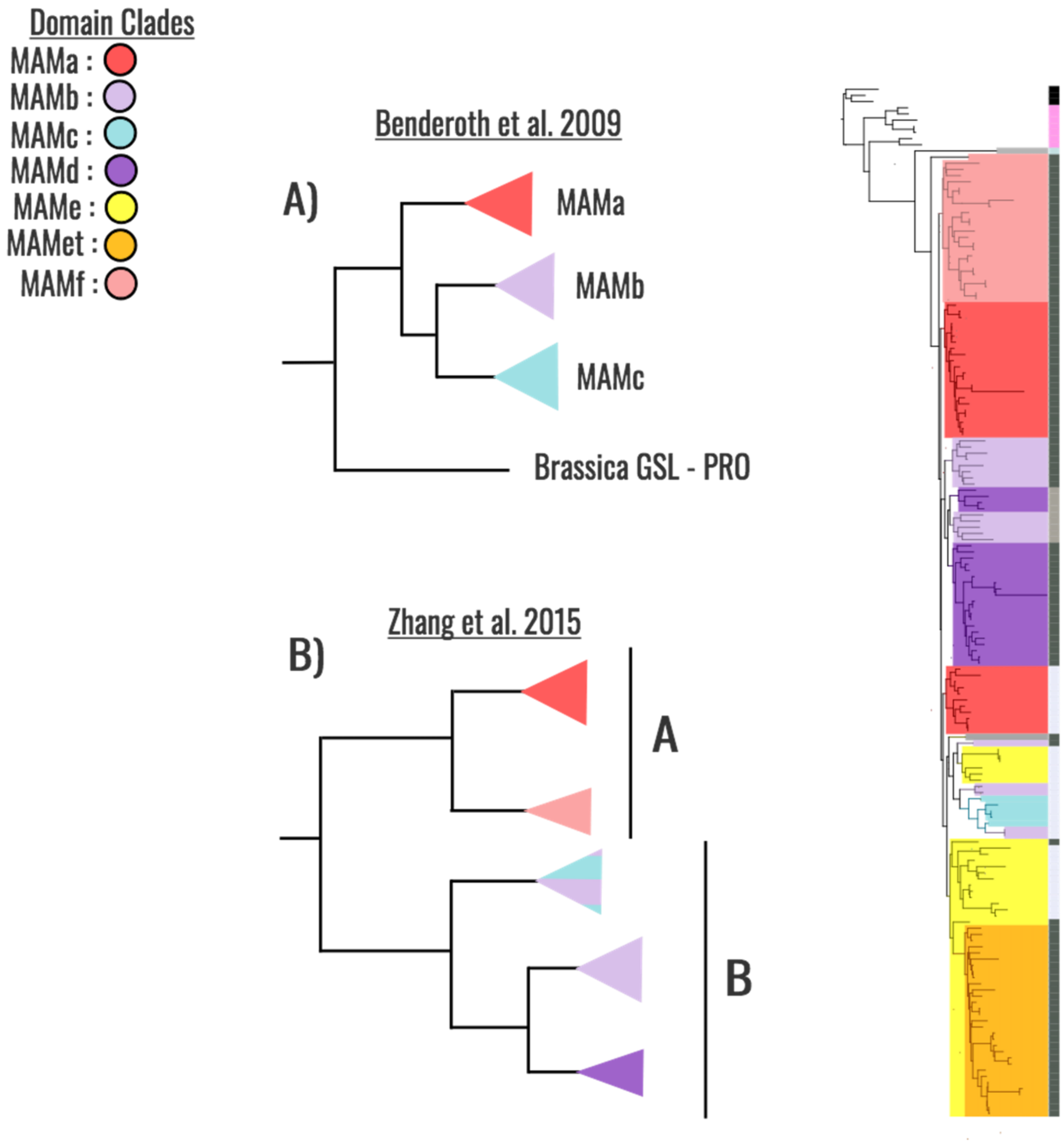

Supplementary Figure 1. (A) Benderoth et al. 2009 describes the MAM lineage in terms of orthology to *Arabidopsis lyrata* gene tree clades. While the topology generally agrees with our tree, the emphasis on Arabidopsis and close relatives gives a limited picture of MAM diversity. This tree also supported the hypothesis that MAM has evolved separately in the Lineage I and II. (B) Zhang et al. 2015 generally agrees with this hypothesis though they do show shared clades not solely informed by the species tree. Some of their topology conflicts with our full sequence tree and yet agrees with the domain specific tree. This may be due to how their alignment was cleaned and their species sampling.

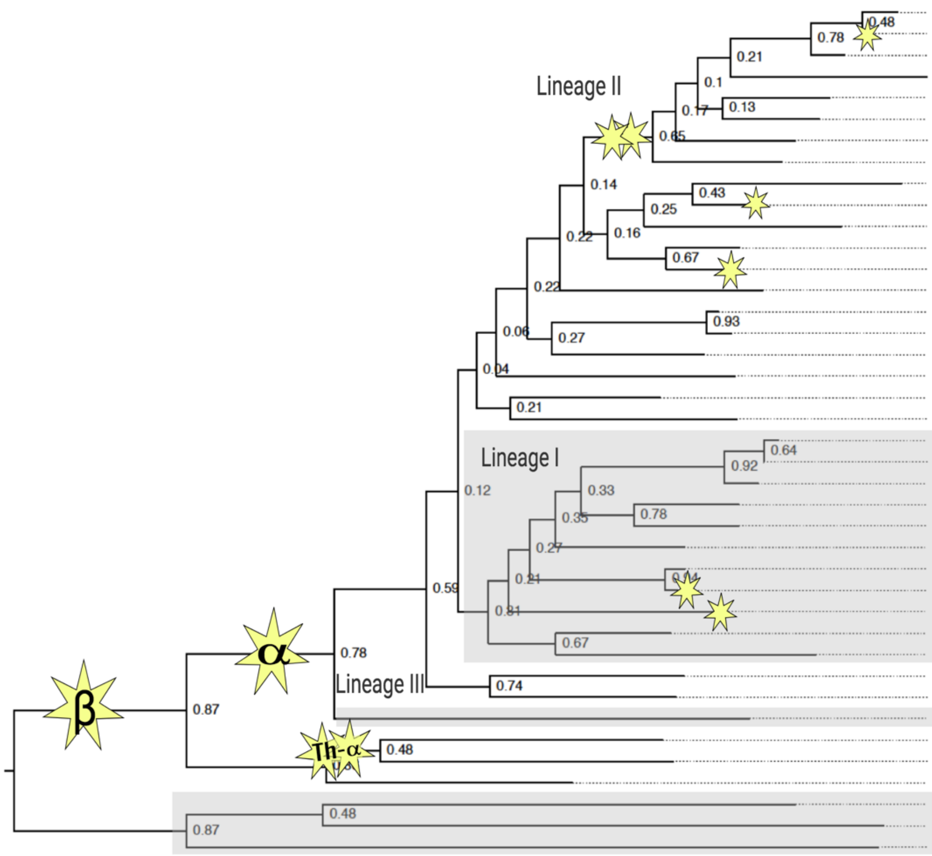

	Overall Gene #	Total IPMS Genes	Total MAM Genes	N of MAM-Anc. Loci	N of Anc. Genes	N of MAM-Tran. Loci	N of Tran. Genes	Transpositions/Uncertain
Brassica oleracea	11	2	9	2	3	1	1	5
Brassica napus	21	4	17	5	7	3	4	6
Brassica rapa	8	2	6	2	3	1	2	1
Raphanus raphanistrum	6	1	5	0	0	0	0	5
Sinapis alba	7	2	5	3	5	0	0	0
Crambe hispanica	6	2	4	2	3	1	1	0
Eruca vesicaria	8	3	5	4	4	1	1	0
Cakile maritima	10	4	6	2	2	0	0	4
Stanleya pinnata	8	3	5	0	0	1	1	4
Caulanthus amplexicaulis	9	4	5	2	3	2	2	0
Sisymbrium irio	3	2	1	0	0	1	1	0
Myagrum perfoliatum	6	2	4	1	3	1	1	0
Isatis tinctoria	14	6	8	1	1	3	3	4
Schrenkiella parvula	5	2	3	1	2	1	1	0
Thellungiella halophila	7	2	5	1	1	0	0	4
Thellungiella salsuginea	5	2	3	1	2	0	0	1
Thlaspi arvense	4	2	2	1	2	0	0	0
Arabis alpina	10	2	8		3	0	0	5
Lunaria annua	6	2	4	2	4	0	0	0
Iberis amara	5	2	3		0	0	0	3
Capsella rubella2	7	2	5	1	3	0	0	2
~~Capsella rubella~~								
Capsella grandiflora	5	2	3	1	2	0	0	1
Arabidopsis lyrata	5	2	3	1	1	0	0	2
Arabidopsis thaliana	4	2	2	1	2	0	0	0
Malcolmia maritima	3	2	1	1	1	0	0	0
Descurainia sophiades	5	2	3	1	3	0	0	0
Descurainia pinnata	10	4	6	3	3	0	0	3
Lepidium sativum	6	4	2	2	2	0	0	0
Rorippa islandica	7	2	5	1	2	0	0	3
Leavenworthia alabamica	6	2	4	1	0	0	0	4
Diptychorcarpus strictus	7	2	5	1	2	0	0	3
Euclidium syriacum	6	2	4	1	1	0	0	3
Aethionema arabicum	3	2	1	1	1	0	0	0
Tarenaya hassleriana	5	2	3	1	2	0	0	1
Cleome gynandra	4	2	2	1	2	0	0	0
Cleome violacea	4	1	3	1	3	0	0	0
Theobroma cacao	1	1	0	0	0	0	0	0
Citrus sinensis	1	1	0	0	0	0	0	0
Vitis vinifera	1	1	0	0	0	0	0	0

Supplementary Figure 2. The overall gene counts per genome for the *MAM/IPMS* gene family. Gene numbers, especially in IPMS, are correlated with recent polyploidy. Three genomes conflict with the expected *IPMS* dosage expectation of multiples of two. The *Raphanus raphanistrum* and *Stanleya pinnata* IPMS deviations may be an artifact of lower quality genomes, but the Eruca vesicaria retention appears to be a newly sub-functionalized *IPMS* copy, exhibiting an intermediate syntenic relationships to that of some *MAM*-Ancestral genes in the Cleomaceae. For MAM, the number of Loci indicates whether MAM-Ancestral or MAM-Transposed has experienced a context duplication. The number of genes at that locus is the overall total of genes across all syntenic loci of that type.

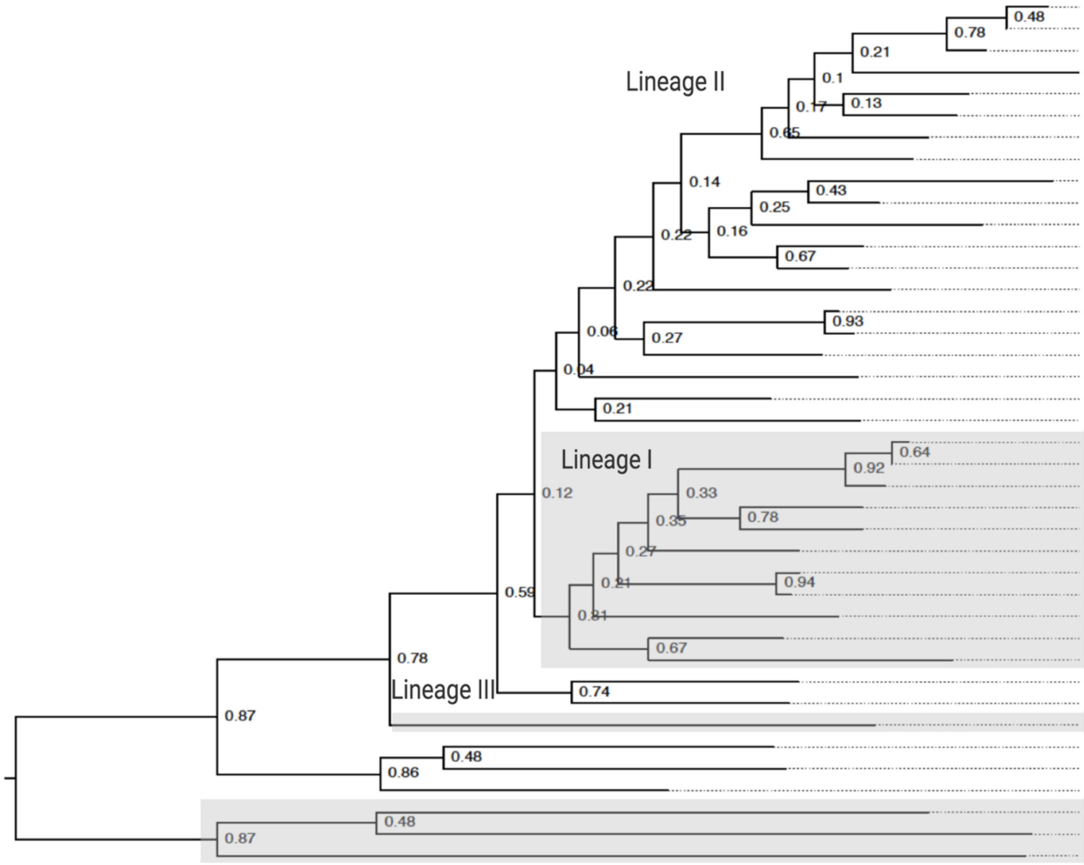

	MAMa	MAMb	MAMc	MAMd	MAMe	MAMet	MAMf	MAM-NA
Brassica oleracea	2	0	0	2	0	3	2	0
Brassica napus	3	0	0	4	0	6	4	0
Brassica rapa	0	0	0	1	0	3	2	0
Raphanus raphanistrum	1	0	0	2	0	2	0	0
Sinapis alba	2	0	0	1	0	0	2	0
Crambe hispanica	1	0	0	1	0	1	1	0
Eruca vesicaria	2	0	0	2	0	1	0	0
Cakile maritima	1	0	0	2	0	2	1	0
Stanleya pinnata	0	0	0	1	0	3	1	0
Caulanthus amplexicaulis	1	0	0	1	0	2	1	0
Sisymbrium irio	0	0	0	0	0	1	0	0
Myagrum perfoliatum	1	0	0	1	0	1	1	0
Isatis tinctoria	1	0	0	1	0	4	2	0
Schrenkiella parvula	0	0	0	1	0	1	1	0
Thellungiella halophila	3	0	0	0	0	0	2	0
Thellungiella salsuginea	1	0	0	0	0	0	2	0
Thlaspi arvense	1	0	0	0	0	0	1	0
Arabis alpina	0	8	0	0	0	0	0	0
Lunaria annua	0	1	0	0	2	0	0	1
Iberis amara	2	0	0	0	0	0	1	0
Capsella rubella2	1	1	2	0	1	0	0	0
~~Capsella rubella~~								
Capsella grandiflora	1	1	1	0	0	0	0	0
Arabidopsis lyrata	1	1	1	0	0	0	0	0
Arabidopsis thaliana	1	1	0	0	0	0	0	0
Malcolmia maritima	1	0	0	0	0	0	0	0
Descurainia sophiades	1	0	0	0	2	0	0	0
Descurainia pinnata	2	0	0	0	4	0	0	0
Lepidium sativum	0	0	0	0	2	0	0	0
Rorippa islandica	1	0	0	0	4	0	0	0
Leavenworthia alabamica	1	0	0	0	3	0	0	0
Diptychorcarpus strictus	0	2	0	3	0	0	0	0
Euclidium syriacum	0	3	0	1	0	0	0	0
Aethionema arabicum	0	0	0	0	0	0	0	1
Tarenaya hassleriana	0	0	0	0	0	0	0	3
Cleome gynandra	0	0	0	0	0	0	0	2
Cleome violacea	0	0	0	0	0	0	0	3
Theobroma cacao	0	0	0	0	0	0	0	0
Citrus sinensis	0	0	0	0	0	0	0	0
Vitis vinifera	0	0	0	0	0	0	0	0

Supplementary Figure 3. Here we show the full domain clade distribution of MAM genes across the genomes, regardless of synteny or genomic position. This data was used ultimately to place the points of innovation for different MAM types in Figure 4.

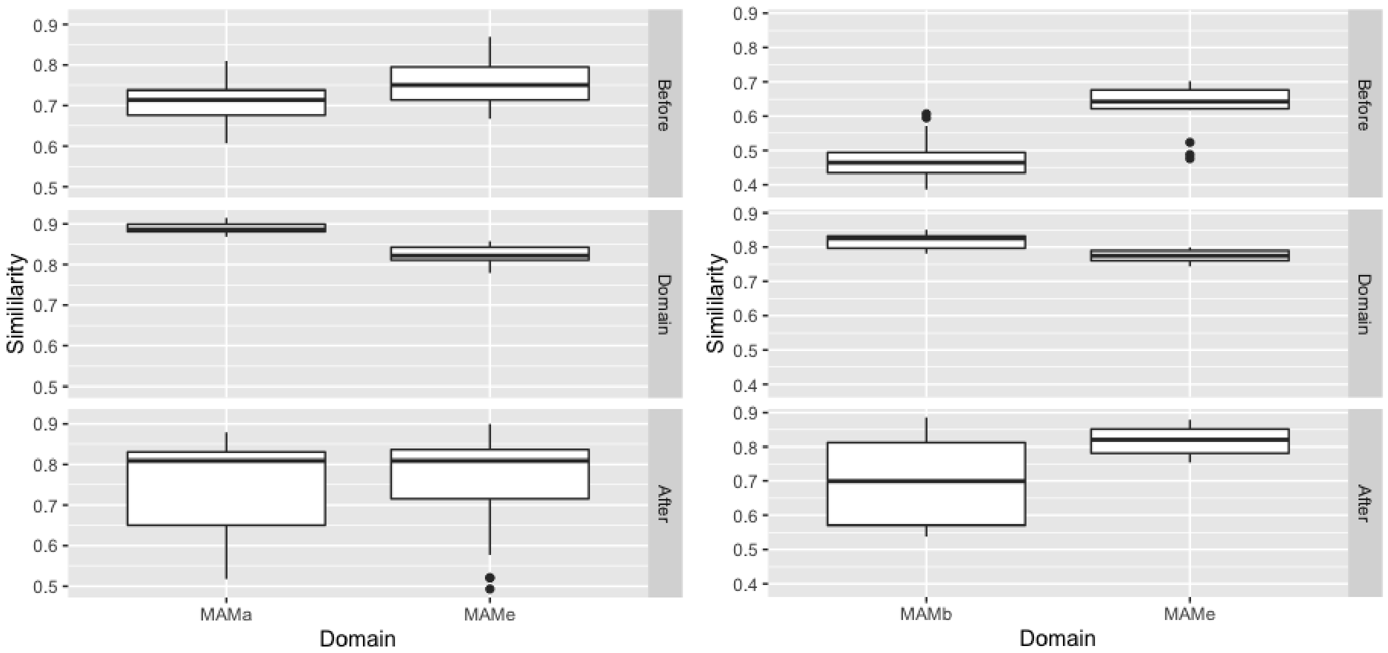

Supplementary Figure 4. MAM protein sequences were divided into before domain, domain, and after domain segments and each significantly different section of the MAMa or MAMb genes from lineage I were compared to corresponding MAMe sections.

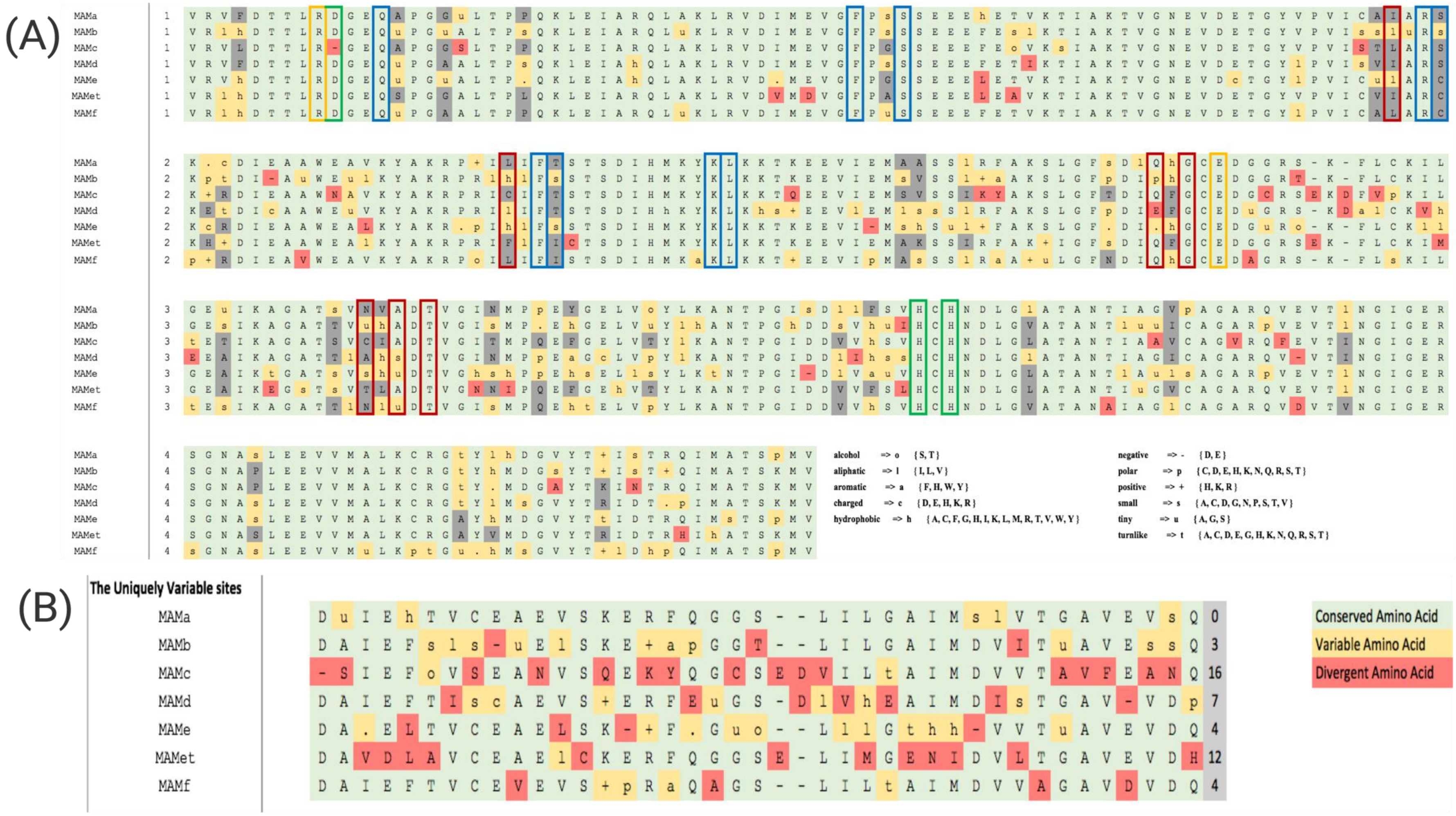

Supplementary Figure 5. Amino acid sequence comparisons at 80% sequence similarity. (A) Colored rectangles indicate specific biochemical functions as described by Kumar et al. 2019 in *Brassica juncea*. Green - metal binding sites; Yellow - catalytic sites; Red - 2-

oxo acid binding sites; Blue - CoA binding sites. (B) Summarizes all sites with a uniquely divergent amino acid to quantify the significance of domain divergence.
