# Supplementary figures and images for: Genomic Origin and Diversification of the Glucosinolate MAM Locus
Source: Front Plant Sci. 2020 Jun 4;11:711. doi: 10.3389/fpls.2020.00711 (PMC7289053; doi:10.3389/fpls.2020.00711)

Tree scale:

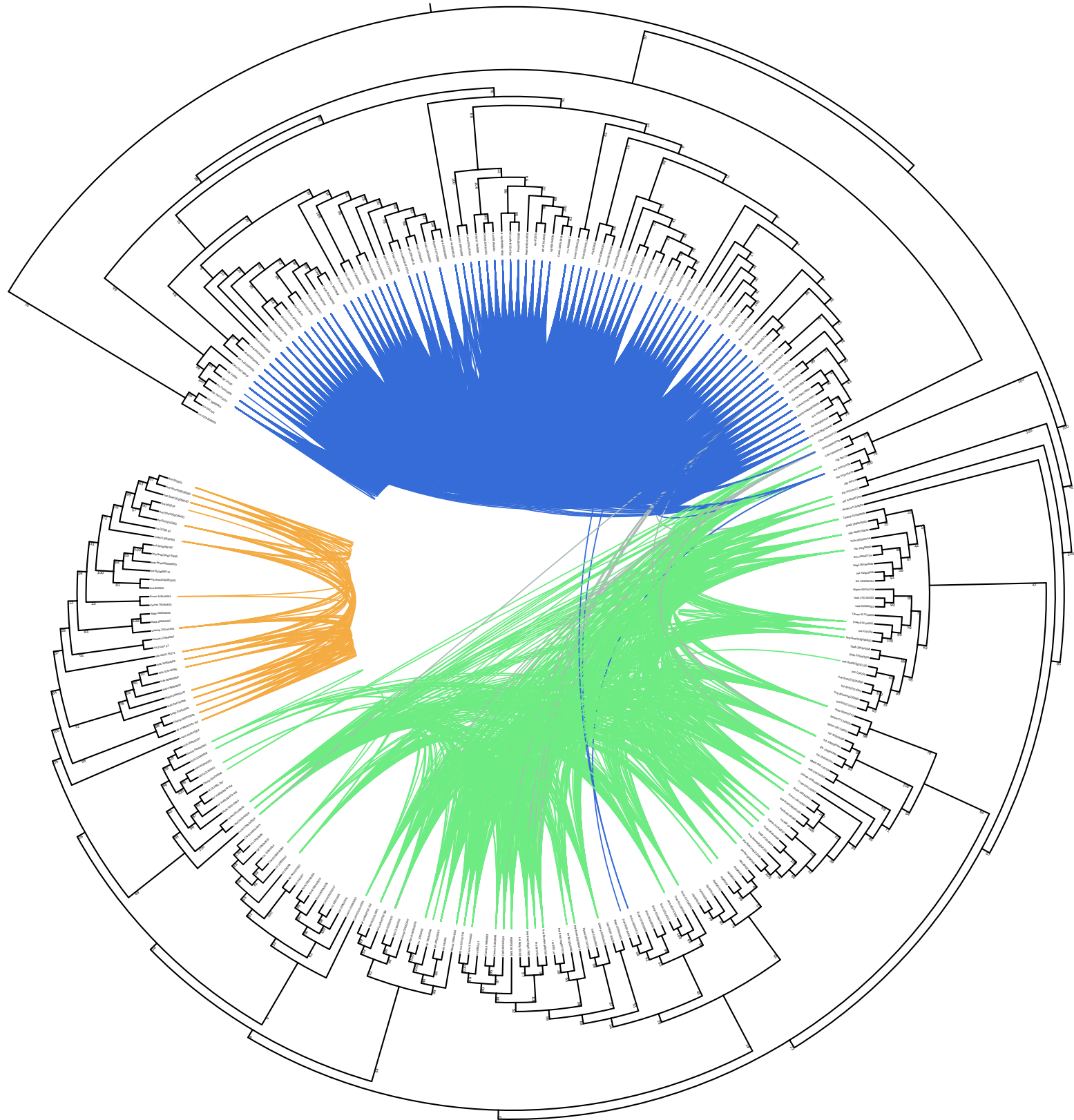

Supplement: FIGURE S6 — Full gene family phylogeny with bootstrap scores at 1000 bootstraps with syntenic clusters mapped. Used in Figure 1. May also be accessed via: http://bit.ly/2tHVgYK. [file Data_Sheet_1.PDF]
