# Supplementary material for: Genomic Origin and Diversification of the Glucosinolate MAM Locus
Source: Front Plant Sci. 2020 Jun 4;11:711. doi: 10.3389/fpls.2020.00711 (PMC7289053; doi:10.3389/fpls.2020.00711)
Supplement: FIGURE S7 — Domain tree phylogeny with clades colored and bootstrap scores at 1000 bootstraps. Used in Figure 3. May also be accessed via: http://bit.ly/2Hb5jIS. [file Data_Sheet_2.PDF]

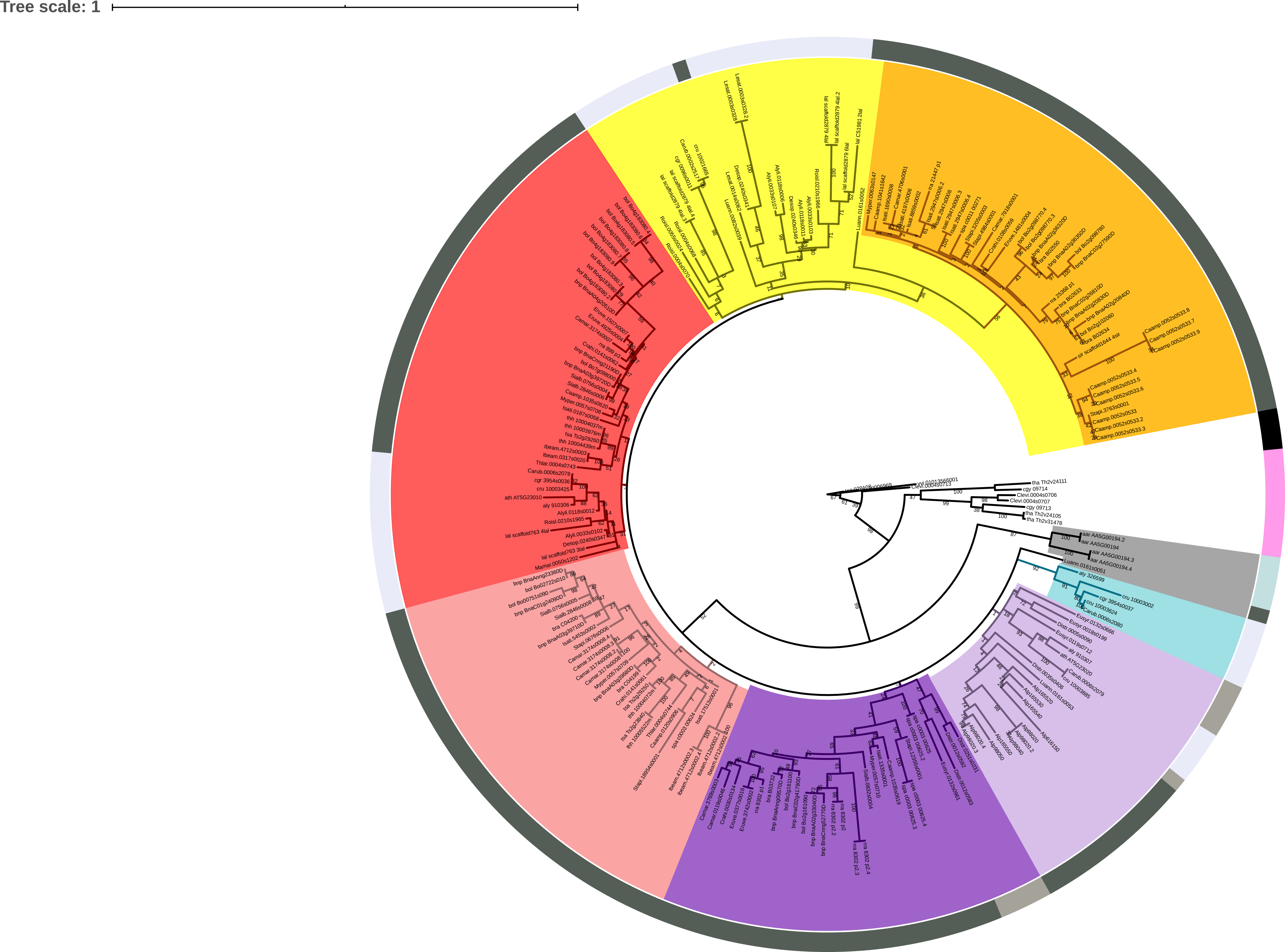
Tree scale: 1
