# Supplementary material for: Genomic Origin and Diversification of the Glucosinolate MAM Locus
Source: Front Plant Sci. 2020 Jun 4;11:711. doi: 10.3389/fpls.2020.00711 (PMC7289053; doi:10.3389/fpls.2020.00711)
Supplement: FIGURE S8 — Full gene family phylogeny with bootstrap scores at 1000 bootstraps with clades colored. Used in Figure 3. May also be accessed via: http://bit.ly/37btHEZ. [file Data_Sheet_3.PDF]

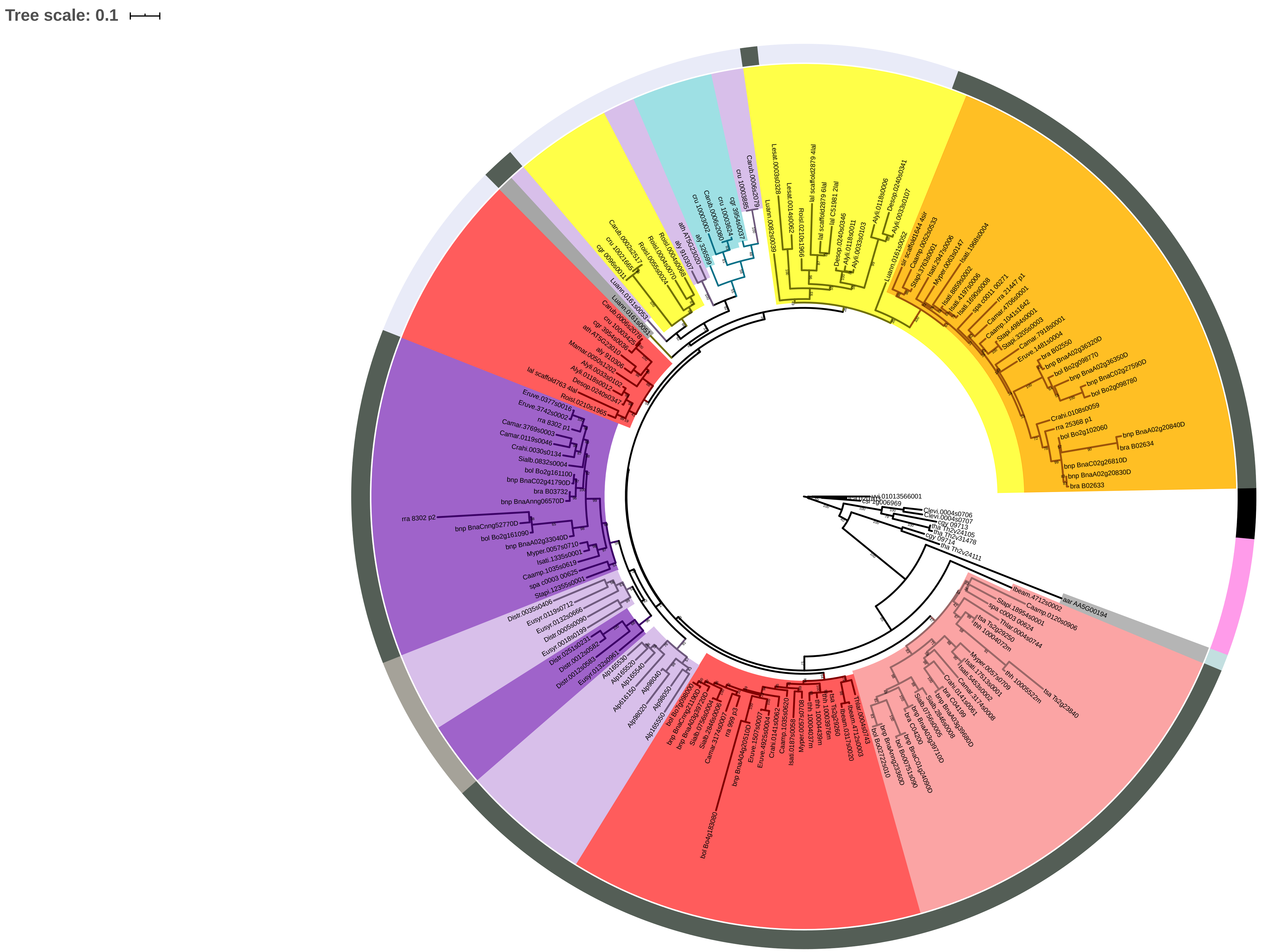
Tree scale: 0.1
